# Landscape of *BCL2* Resistance Mutations in a Real-World Cohort of Patients with Relapsed/Refractory Chronic Lymphocytic Leukemia Treated with Venetoclax

**DOI:** 10.3390/ijms24065802

**Published:** 2023-03-18

**Authors:** Lili Kotmayer, Tamás László, Gábor Mikala, Richárd Kiss, Luca Lévay, Lajos László Hegyi, Stefánia Gróf, Tibor Nagy, Gábor Barna, Péter Farkas, Júlia Weisinger, Zsolt Nagy, Alexandra Balogh, Tamás Masszi, Judit Demeter, Adrienn Sulák, Zoltán Kohl, Hussain Alizadeh, Miklós Egyed, Piroska Pettendi, Lajos Gergely, Márk Plander, Zsolt Pauker, András Masszi, András Matolcsy, Róbert Szász, Csaba Bödör, Donát Alpár

**Affiliations:** 1 HCEMM-SE Molecular Oncohematology Research Group, Department of Pathology and Experimental Cancer Research, Semmelweis University, 1085 Budapest, Hungary; 2 South-Pest Central Hospital, National Institute of Hematology and Infectology, 1097 Budapest, Hungary; 3 Department of Biochemistry and Molecular Biology, Faculty of Medicine, University of Debrecen, 4032 Debrecen, Hungary; 4 Department of Internal Medicine and Hematology, Semmelweis University, 1085 Budapest, Hungary; 5 Department of Internal Medicine and Oncology, Semmelweis University, 1085 Budapest, Hungary; 6 2nd Department of Internal Medicine and Cardiology Center, University of Szeged, 6725 Szeged, Hungary; 7 1st Department of Internal Medicine, Clinical Centre, University of Pécs, 7622 Pécs, Hungary; 8 Kaposi Mór University Teaching Hospital of County Somogy, 7400 Kaposvár, Hungary; 9 Hetényi Géza Hospital, Clinic of County Jász-Nagykun-Szolnok, 5000 Szolnok, Hungary; 10 Division of Hematology, Department of Internal Medicine, University of Debrecen, 4032 Debrecen, Hungary; 11 Markusovszky University Teaching Hospital, 9700 Szombathely, Hungary; 12 Borsod-Abaúj-Zemplén County Hospital and University Teaching Hospital, 3515 Miskolc, Hungary; 13 National Institute of Oncology, 1122 Budapest, Hungary; 14 Department of Laboratory Medicine, Karolinska Institute, 171 77 Solna, Sweden

**Keywords:** CLL, venetoclax, BCL2, therapy resistance, molecular monitoring

## Abstract

The oral, highly selective Bcl2 inhibitor venetoclax has substantially improved the therapeutic landscape of chronic lymphocytic leukemia (CLL). Despite the remarkable response rates in patients with relapsed/refractory (R/R) disease, acquired resistance is the leading cause of treatment failure, with somatic *BCL2* mutations being the predominant genetic drivers underpinning venetoclax resistance. To assess the correlation between disease progression and the most common *BCL2* mutations G101V and D103Y, sensitive (10^−4^) screening for the most common *BCL2* mutations G101V and D103Y was performed in 67 R/R CLL patients during venetoclax single-agent or venetoclax–rituximab combination therapy. With a median follow-up time of 23 months, *BCL2* G101V and D103Y were detected in 10.4% (7/67) and 11.9% (8/67) of the cases, respectively, with four patients harboring both resistance mutations. Ten out of eleven patients carrying *BCL2* G101V and/or D103Y experienced relapse during the follow-up period, representing 43.5% of the cases (10/23) showing clinical signs of disease progression. All *BCL2* G101V or D103Y variants were detected in patients receiving venetoclax as a continuous single-agent treatment while these mutations were not observed during or after fixed-duration venetoclax therapy. Targeted ultra-deep sequencing of *BCL2* uncovered three additional variants in four patient samples obtained at relapse, suggesting convergent evolution and implying a cooperating role of *BCL2* mutations in driving venetoclax resistance. This cohort is the largest R/R CLL patient population reported to date in which *BCL2* resistance mutations were investigated. Our study demonstrates the feasibility and clinical value of sensitive screening for *BCL2* resistance mutations in R/R CLL.

## 1. Introduction

Clinical outcomes of chronic lymphocytic leukemia (CLL) have significantly improved over the past 10 years with targeted therapies changing the treatment paradigm of the disease. Remarkable outcomes in first-line as well as in relapsed/therapy refractory (R/R) CLL are observed in patients with coexisting conditions, as well as in patient populations characterized by high-risk disease biology, including deletions of 11q and 17p, *TP53* mutations, and unmutated IGHV status [1,2,3,4]. Venetoclax, a first-in-class BH3 mimetic agent inhibiting the pro-survival Bcl2 is approved in both front-line and relapsed CLL, delivering remarkable results with complete response rates of up to 30–50% [5,6,7,8]. Current treatment options include continuous venetoclax monotherapy or time-limited therapy for one year in combination with obinutuzumab in frontline cases or for two years in combination with rituximab in patients with prior lines of therapy [3,6,7,9].

Despite the high response rates and prolonged progression-free survival, loss of efficacy and subsequent secondary venetoclax resistance occur in a subset of patients, in both continuous and fixed-duration treatment setups [5,6]. Over the past few years, heterogeneous molecular mechanisms of resistance have been observed in vitro as well as under clinical circumstances. The spectrum of molecular changes reported to be associated with acquired resistance includes alterations in the pro-survival gene *BCL2* diminishing venetoclax binding, overexpression of the anti-apoptotic BCL-XL, Bcl-2, MCL1, and XIAP, and the emergence of complex karyotypes [10,11,12,13,14,15,16,17]. Mitochondrial metabolic reprogramming has also been reported as a putative driver mechanism of venetoclax resistance through AMPK-regulated oxidative phosphorylation in patients with refractory CLL [16,18].

A handful of mutations affecting the *BCL2* gene have been demonstrated to confer resistance to venetoclax by reducing or inhibiting the binding affinity of the small molecule inhibitor for the anti-apoptotic Bcl2 protein. The glycine-to-valine substitution emerging at position G101 (G101V) was first reported by Blombery et al. in seven patients showing progressive disease during venetoclax treatment [13]. *BCL2* G101V mitigates the cellular response to venetoclax in vitro via modulation of the BH3 binding domain of Bcl2 and is associated with clinical resistance in up to 50% of the patients experiencing relapse or disease progression [13,14,19]. Over the past few years, several additional *BCL2* mutations, e.g., D103Y directly disrupting the BH3 binding P4 pocket, have been observed in CLL patients, unveiling various cooperating mutational patterns with coexisting mutations and overexpression of the pro-survival proteins BCL-XL and MCL1 [13,14,20,21]. Although the proportion of CLL cells carrying resistance-associated molecular changes may vary widely from minor subclones to large CLL compartments, subclonal presence and cumulative abundance of genetically and/or transcriptionally altered leukemic cells are reported in the majority of cases [12,13,20]. Intriguingly, subclonality of mutation-bearing or BCL-XL/MCL1-overexpressing CLL cells has been observed even in samples obtained at the time of relapse or disease progression, implying a significant role of the permissive microenvironment and cooperating genetic and epigenetic events in driving acquired venetoclax resistance leading to overt clinical relapse [12,13,14,20,22,23].

In this study, we have assessed the clinical value and feasibility of sensitive screening for the two most common *BCL2* resistance mutations, G101V and D103Y, in a cohort of patients with R/R CLL outside of clinical trials receiving single-agent or venetoclax plus rituximab combination therapy. Our results provide an insight into the frequency of these variants in patients progressing on venetoclax and into the repertoire of additional, simultaneously detectable *BCL2* mutations. Our data on *BCL2* G101V and D103Y may also have implications for the sensitive monitoring of venetoclax therapy which may allow for the early detection of an impending relapse and the identification of patients who could potentially benefit from an alternative approach to therapeutic intervention.

## 2. Results

### 2.1. Molecular and Cytogenetic Characteristics of the Study Cohort

Molecular and cytogenetic aberrations considered independent prognostic and/or predictive markers in CLL were screened as part of the standard-of-care diagnostic workflow (Supplementary Table S1). Del(17p) status analyzed by FISH and *TP53* mutation positivity screened by targeted NGS were available in 94.0% (63/67) and 83.6% (56/67) of the cases, respectively. Patients harboring del(17p) and/or *TP53* mutation(s) comprised 40.3% (27/67) of the patient population. Additional cytogenetic aberrations including del(11q), del(13q), and trisomy of chromosome 12 were identified in 22.4% (15/67), 22.4% (15/67), and 11.9% (8/67) of the cases, respectively. IGHV mutation status was assessed in 52 patients, with 82.7% (43/52) of them carrying unmutated IGHV (IGHV-U). Patients with high-risk genetic features associated with adverse prognosis including *TP53* aberrations, and IGHV-U status represented 83.6% (56/67) of the study cohort, which together with the failure of prior therapy lines depicted an R/R patient population typically selected for venetoclax therapy.

### 2.2. Sensitive Screening for BCL2 G101V and D103Y

**Figure 1:**
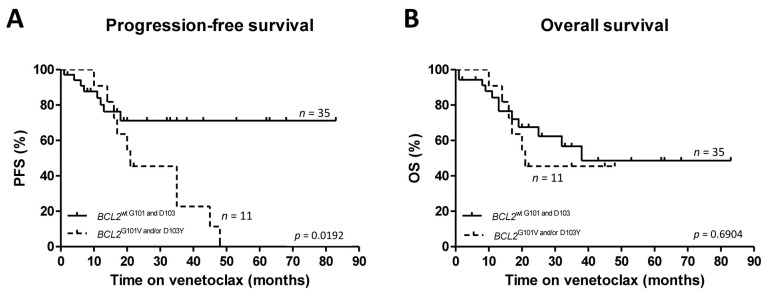
Progression-free survival (PFS) and overall survival (OS) of relapsed/refractory CLL patients receiving continuous venetoclax therapy harboring *BCL2* G101V and/or D103Y mutations. (**A**) PFS was significantly shorter in patients with *BCL2* G101V and/or D103Y compared to *BCL2* G101 and D103 wild-type cases. Data were censored at the time of the last follow-up in patients not experiencing disease progression. (**B**) While a trend toward shorter OS was also observed among patients with *BCL2* G101V and/or D103Y, the difference between the two patient populations did not reach statistical significance.

Screening for *BCL2* G101V and D103Y mutations was performed on samples obtained from 67 R/R patients using locus-specific ddPCR assays designed and optimized for the sensitive (10^−4^) interrogation of these two mutational hotspots. *BCL2* G101V and D103Y were detected in 10.4% (7/67) and 11.9% (8/67) of the cases, respectively, with 4 patients carrying both alterations. The two variants were detected after a median of 15 months (range: 5–48 months) of venetoclax therapy with 90.9% (10/11) of the affected patients experiencing relapse or disease progression. These ten patients represented 43.5% (10/23) of all cases developing secondary venetoclax resistance during the follow-up period. Richter’s transformation was observed in one patient who tested positive for *BCL2* G101V and in another patient with wild-type G101 and D103 alleles after 15 and 25 months of venetoclax therapy, respectively. *BCL2* G101V and D103Y were exclusively detected in patients with continuous venetoclax therapy, and thus cases with a time-limited treatment setup were excluded from the PFS and OS analyses. In the analyzed patient population, compared to cases with wild-type *BCL2* G101 and D103 loci, significantly inferior PFS as defined by the iwCLL 2018 criteria [24] was observed among patients carrying *BCL2* G101V and/or D103Y mutations (Mantel–Cox test, *p* = 0.0192) (Figure 1A), with the latter subgroup showing a median PFS of 21 months (range: 3–48 months). No significant difference in overall survival rates was observed between the two patient populations with subsequent lines of therapy applied in both cohorts (Mantel–Cox test, *p* = 0.6904) (Figure 1B). The *TP53* status and number of prior therapy lines did not influence the PFS in patients with continuous venetoclax monotherapy (Supplementary Figure S1). Intriguingly, all *BCL2* G101V or D103Y variants were detected in patients receiving venetoclax as a continuous single-agent treatment while these two mutations were not observed during or after fixed-duration venetoclax therapy. Median CCF values of G101V and D103Y at the time of sample collection were 0.29% (range: 0.047–7.8%) and 0.28% (range: 0.018–8.94%), respectively. The median CCF value of *BCL2* D103Y at the time of sample collection in four cases with wild-type G101 was 0.023% (range: 0.015–0.16%).

### 2.3. Management of Secondary Venetoclax Resistance in Patients with G101V and/or D103Y

**Figure 2:**
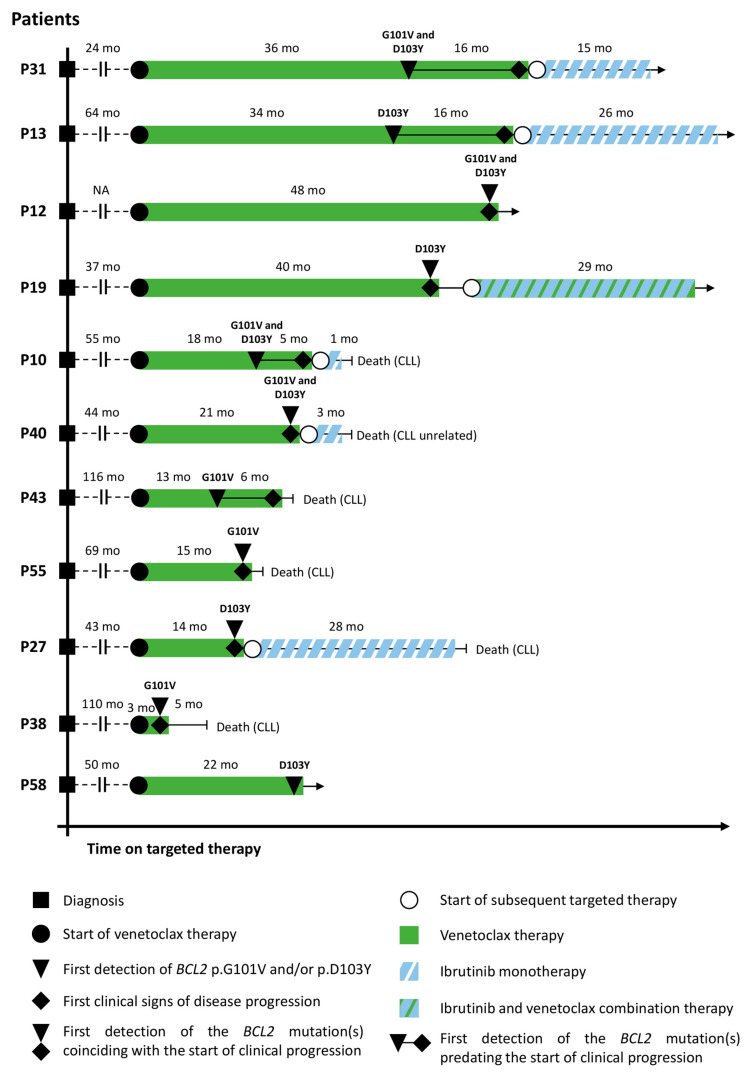
Treatment timeline of 11 patients treated with venetoclax and harboring detectable *BCL2* G101V and/or D103Y mutations. A total of 10 out of 11 patients experienced relapse or disease progression during the follow-up period with 6 of them receiving subsequent ibrutinib monotherapy or salvage ibrutinib plus venetoclax combination. In 4 cases with samples obtained prior to disease progression, the emergence of the *BCL2* G101V and/or D103Y predated the first clinical signs of relapse with a median of 11 months (range: 6–16 months). Only 1 patient (P61) carrying *BCL2* D103Y is still alive to date without showing any clinical signs of disease progression after 22 months of venetoclax therapy. CCF = cancer cell fraction.

Due to secondary venetoclax resistance and subsequent disease progression, 10/11 patients harboring *BCL2* G101V and/or D1013Y discontinued single-agent venetoclax after a median of 21 months (range: 10–48 months) of Bcl2 inhibition (Figure 2). Subsequent BTK inhibitor ibrutinib therapy was administered in 60% (6/10) of the cases with one patient receiving ibrutinib in combination with continued venetoclax treatment. Despite the temporary regression in clinical symptoms across the cases, three patients receiving salvage ibrutinib experienced relapse and developed secondary ibrutinib resistance after a median of 28 months (range: 1–29 months) of BTK inhibition, with two out of six patients having succumbed to their disease. One patient developing detectable *BCL2* G101V and D1013Y mutations on venetoclax and receiving subsequent ibrutinib therapy died from reasons unrelated to CLL during the follow-up period. Three out of ten Bcl-2 mutant patients receiving venetoclax and experiencing disease progression died without receiving any subsequent therapy, with one out of the three patients undergoing Richter’s transformation. Only 1 patient carrying *BCL2* D103Y is still alive to date with no clinical signs of disease progression following 22 months of continuous venetoclax monotherapy. Follow-up was terminated and data were locked on 6 May 2022.

### 2.4. Additional BCL2 Mutations Revealed by Targeted Next-Generation Sequencing

Ultra-deep targeted NGS was performed on 17 samples obtained at the time of disease progression, irrespective of their *BCL2* G101V and D103Y mutation status assessed by ddPCR. The investigated cohort comprised 8 cases with *BCL2* G101V and/or D103Y, and a subset of patients (9 cases) with wild-type G101 and D103 developing secondary venetoclax resistance after a median of 15 months (range: 1–48 months) of venetoclax therapy. Targeted ultra-deep sequencing was performed covering all coding regions of the *BTK*, *PLCG2*, *BCL2,* and *TP53* genes with a median depth of 3886× (range: 942–10,079×). NGS uncovered three additional *BCL2* mutations, namely, D103E, A113G, and V156D in two cases. Notably, both patients harbored G101V and/or D103Y as previously assessed by ddPCR, with no *BCL2* mutations unveiled in *BCL2* G101 and D103 wild-type cases.

Considering *TP53* variants unveiled by targeted NGS, two patients (patients #25 and #40) acquired detectable TP53 mutations during venetoclax therapy, with these aberrations being exclusively observed in samples obtained at the time of disease progression and not in diagnostic specimens. Interestingly, one patient (patient #43) was found to be *TP53* wild-type at progression, while harboring a somatic *TP53* variant at the time of diagnosis. Further somatic variants uncovered by ultra-deep NGS in samples obtained at the time of disease progression are listed in Supplementary Table S2.

### 2.5. Time-Resolved Monitoring of Co-Occurring BCL2 Resistance Mutations

**Figure 3:**
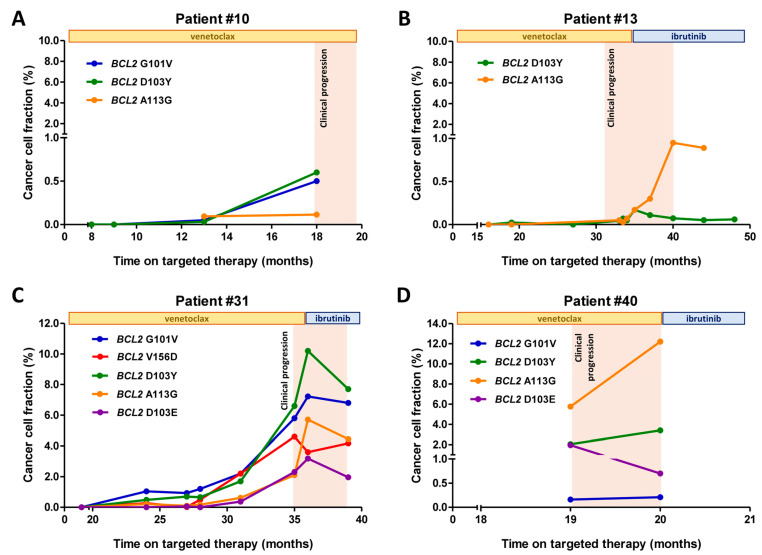
Temporal dynamics of *BCL2* mutations in four patients harboring multiple variants associated with venetoclax resistance. (**A**) In patient #11, *BCL2* G101V and D103Y were first detectable after 13 months of continuous venetoclax monotherapy with fractional abundance (FA) values of 0.05% and 0.03%, respectively. Additionally, a coexistent subclonal A113G variant was identified by ultra-sensitive droplet digital PCR (ddPCR) in this patient. The first detection of the three mutations predated the first clinical signs of disease progression by 5 months. (**B**) Patient #14 harbored *BCL2* D103Y detected by ddPCR with a cancer cell fraction (CCF) value of 0.17%. The aberration was successfully backtracked in samples obtained prior to progression. Additional ultra-sensitive ddPCR uncovered a cooperating A113G mutation in the sample collected following the failure of Bcl2 inhibition. (**C**) In a sample obtained from patient #32 at disease progression following 35 months of continuous venetoclax monotherapy, both *BCL2* G101V and D103Y were detected with CCF values of 5.8% and 6.6%, respectively. NGS analysis of the same sample uncovered three coexisting variants, namely, A113G, D103E, and V156D, with CCF values of 2.1%, 2.3%, and 6.6%, respectively. All variants excluding D103E were successfully backtracked in samples obtained 16 months prior to progression. Interestingly, D103E was first detectable after 27 months of venetoclax therapy, suggesting multi-layered subclonal changes driving secondary venetoclax resistance. Subsequent ibrutinib was administered following the onset of clinical progression which resulted in an observable decline in the allelic burden of all *BCL2* variants. (**D**) Patient #42 harbored four distinct *BCL2* variants, namely, G101V, D103Y, A113G, and D103E, observed in the sample collected at the time of disease progression with CCF values of 0.21%, 3.4%, 12.2%, and 0.7%, respectively.

**Figure 4:**
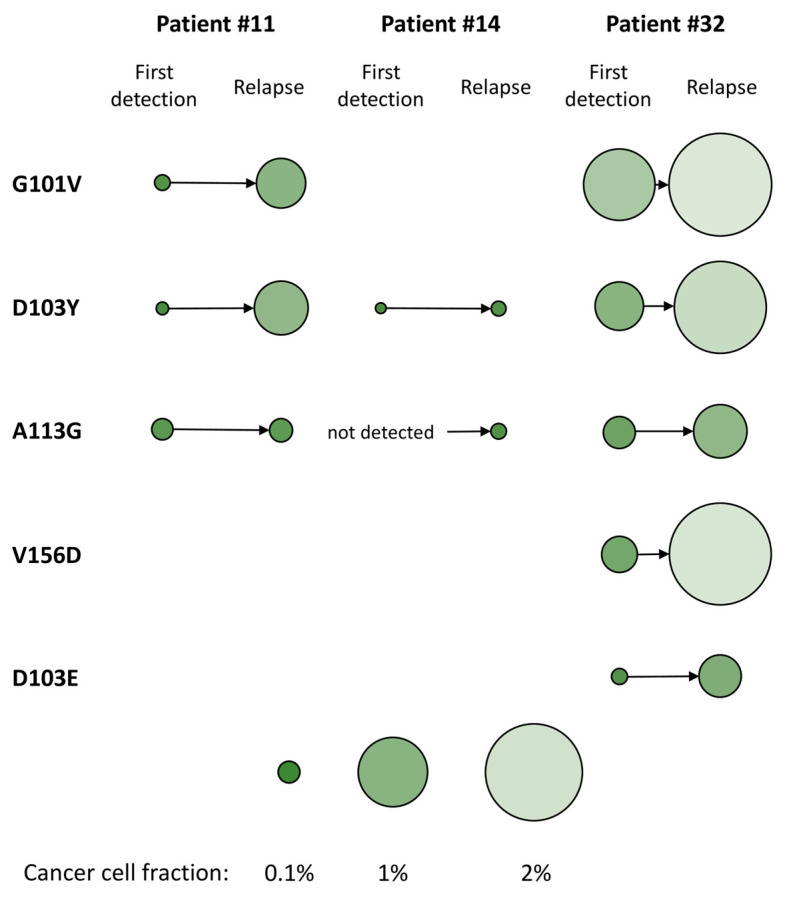
Cancer cell fraction (CCF) values of *BCL2* variants in patients with samples obtained prior to disease progression versus at the time of relapse. CCF values of G101V, D103Y, A113G, V156D, and D103E showed an increasing tendency ubiquitously across the three analyzed patients. Diameter and color of circles are proportional to the allelic burden of each mutation in individual samples. In patient #14, the *BCL2* A113G mutation was first detected in the sample obtained at the time of disease progression.

Four patients harboring multiple *BCL2* mutations experienced relapse or disease progression after a median of 29 months (range: 18–48 months) of continuous single-agent venetoclax therapy (Figure 3). Sensitive ddPCR was performed on longitudinal samples of all four cases allowing for a retrospective temporal dissection of the detected variants using custom-designed assays with sequences specific for the discriminative analysis of mutant and wild-type alleles of *BCL2* D103E, A113G, and V156D (Supplementary Table S3). In samples obtained at the time of disease progression, median CCF values of *BCL2* G101V, D103Y, D103E, A113G, and V156D were 0.5% (range: 0.16–2.20%), 1.15% (range: 0.05–3.40%), 1.16% (range: 0.37–1.95%), 0.37% (range: 0.05–5.76%), and 2.2%, respectively. In 4 patients with samples obtained prior to progression, the emergence of *BCL2* G101V and/or D103Y, as well as co-occurring *BCL2* mutations preceded the first clinical signs of disease progression by a median of 11 months (range: 5–16 months) (Figure 3A–C, patient #43 harboring exclusively G101V mutation is not shown). The allelic burdens of *BCL2* G101V, D103Y, D103E, A113G, and V156D were increased at the time of relapse as compared to samples collected prior to disease progression, demonstrating a median 6.61-fold (range: 1.2–18.75) increase in the CCF values of each mutation over time (Figure 4).

### 2.6. Secondary Venetoclax Resistance in Cases with Wild-Type BCL2

Thirteen patients progressing on venetoclax carried wild-type *BCL2* G101 and D103 as assessed by ddPCR. This subset of patients represented 56.5% of the cases developing secondary venetoclax resistance after a median of 13 months (range: 1–33 months) of Bcl2 inhibition. Interestingly, all patients experiencing relapse or disease progression received continuous single-agent venetoclax therapy. Subsequent salvage R-CHOP chemo-immunotherapy was applied in two cases. In one patient, subsequent rituximab was administered in combination with continued venetoclax and in one case, salvage ibrutinib was added to the continuous venetoclax monotherapy. Eight patients succumbed to their disease after developing venetoclax resistance without receiving any subsequent lines of treatment. As an attempt to unveil alternative molecular mechanisms underlying venetoclax resistance, targeted ultra-deep NGS was performed on samples available at the time of disease progression from nine patients carrying wild-type *BCL2* G101 and D103, as previously described. No further resistance mutation affecting the *BCL2* gene was identified in any of the analyzed cases, suggesting either the presence of alternative genomic lesions localized outside the coding region of *BCL2* or the emergence of epigenomic/transcriptomic changes eventually leading to venetoclax resistance.

## 3. Discussion

Bcl2 inhibition by venetoclax has revolutionized the therapeutic landscape of R/R CLL with high response rates and prolonged PFS in heavily pre-treated patient populations [3,6,25,26]. Despite the remarkable improvement in outcome, progressive disease is still a major clinical concern as loss of efficacy eventually occurs in a subset of patients receiving venetoclax [11,27]. Indeed, since the failure of Bcl2 inhibition confers poor outcomes without additional/subsequent salvage therapy, there is a high clinical demand for predicting relapse or disease progression, which could potentially be addressed by the molecular monitoring of recurrent *BCL2* mutations associated with resistance in longitudinally collected samples of patients undergoing venetoclax treatment.

In the framework of a nationwide effort, we investigated the clinical value and feasibility of a sensitive ‘snapshot’ screening for the two most common *BCL2* mutations associated with acquired venetoclax resistance in a cohort of 67 patients with R/R CLL treated with venetoclax either as monotherapy or in combination with rituximab. Using an ultra-sensitive ddPCR method, *BCL2* G101V and/or D103Y mutations were detected in 16.4% of the analyzed patient population, with some 90% of the affected patients showing clinical signs of relapse during the follow-up period. This subset of *BCL2* G101V and/or D103Y positive cases represented over 40% of the patients undergoing clinical disease progression. To unveil additional cooperating genetic changes driving venetoclax resistance, targeted ultra-deep NGS was performed on samples obtained at the time of disease progression, irrespective of the *BCL2* G101 and D103 status previously assessed by ddPCR. In four patients, NGS uncovered three additional *BCL2* mutations, such as D103E, A113G, and V156D, which were previously found to be co-occurring with G101V and associated with resistance to Bcl2 inhibition in vivo [22,28]. Retrospective analysis and temporal dissection of the uncovered *BCL2* variants in longitudinally collected patient samples revealed a heterogeneous and dynamically changing subclonal abundance of mutations during venetoclax therapy, suggesting a complex underlying architecture of resistant CLL cells, coupled with subsequent clinical disease progression in all four cases. These patients represent the first longitudinally monitored and temporally dissected cases with venetoclax resistance to date.

Our findings are in line with previous studies performed on patient cohorts of various clinical trials. Anderson et al. investigated 67 patients with R/R CLL receiving venetoclax in 3 early-phase trials and identified *BCL2* G101V in 11 cases with all patients ultimately experiencing disease progression [11,20]. Consistent with our observations, the allelic burden of the mutation ranged from 0.1% to 68.7% displaying significant heterogeneity within the analyzed patient population. In the same cohort, Blombery et al., uncovered a median of three additional recurrent *BCL2* mutations, including D103Y, D103E, A113G, and V156D, in all but one patient using digital and hybrid capture-based NGS approaches [20,29]. Similarly, Lucas et al., reported recurrent *BCL2* mutations, including A113G, F104L, F104S, L119V, and G101A coexisting with G101V, and the internal tandem duplication p.Arg107_Arg110, in a cohort of 24 patients with venetoclax and ibrutinib-resistant CLL [21]. These studies, together with our observations of additional acquired variants, provide a growing body of evidence of the branching subclonal composition of venetoclax-resistant tumor compartments in individual patients, and highlight the common presence of multiple, independent mechanisms driving clinical disease progression [12,29].

Among the additionally unraveled *BCL2* mutations, substitutions emerging at the D103 residue are especially noteworthy. D103 residue is located within the P4 pocket of Bcl2 establishing a hydrogen bond with the venetoclax [30]. Amino acid substitutions of D103 impart the affinity of Bcl2 for venetoclax without disrupting the protein’s pro-survival function and its affinity for BH3-only proteins as observed in vitro by Blombery et al. [20]. In the same study, missense mutations D103E, D103V, and D103Y were identified across six patients with R/R CLL. *BCL2* D103Y was first reported by Tausch et al., as a cooperating mutation driving venetoclax resistance in a single patient who previously tested positive for G101V [14]. In our cohort, this aberration was detected in eight patients with four of them carrying D103Y in the absence of G101V. To our knowledge, this is the first report on D103Y underpinning venetoclax resistance as a sole detectable alteration affecting *BCL2*. In addition, here we report recurrent *BCL2* D103E co-occurring with other changes of *BCL2* in two patients, further underlining the role of D103 residue in driving venetoclax resistance.

All cases harboring multiple *BCL2* variants displayed cooperating subclonal mutational patterns with allelic burdens of less than 10%, even in samples obtained at the time of relapse. Plausible explanations for this phenomenon may include the key role of mutually exclusive mutation-bearing subclones reported by Thompson et al., the permissive microenvironment as well as the ability of mutation-bearing cells to promote resistance in *BCL2* unmutated CLL subclones [12,20,29,31]. Nevertheless, these assumptions should be further strengthened by supporting data from functional studies in order to comprehensively characterize alternative mechanisms of venetoclax resistance driving progression in distinct CLL compartments. Notably, in our cohort, all patients harboring detectable *BCL2* mutations and experiencing clinical disease progression could have been identified with an exclusive screening for G101V and D103Y, demonstrating the potential clinical value of a standard-of-care ddPCR test targeting these two variants only for the early identification of venetoclax-resistant cases in the future. Intriguingly, no *BCL2* mutation was observed among patients undergoing time-limited venetoclax therapy either as a single agent or in combination with rituximab. This finding is in line with the results of previous studies including an evaluation of the 4-year data of the phase 3 MURANO trial by Kater et al. [6,32]. The authors analyzed end-of-treatment samples from 194 patients receiving fixed-duration venetoclax plus rituximab combination therapy and observed recurrent mutations in the *BIRC3*, *BRAF*, *TP53*, *NOTCH1,* and *XPO1* genes, with no variants identified in *BCL2* [32].

The significance of detecting resistance to Bcl2 inhibition early on in both time-limited and continuous treatment settings has been increasing with the growing number of alternative therapeutic options available for patients developing venetoclax resistance [33,34,35,36]. Distinct genomic and transcriptomic mechanisms conferring resistance to targeted agents allow for adequate disease control using alternative classes of targeted drugs, such as the Bruton tyrosine kinase inhibitors (BTKi). Subsequent covalent BTK inhibition by ibrutinib was applied in 60% of the cases carrying *BCL2* mutations and experiencing relapse or disease progression in our cohort, with 50% of these patients delivering durable responses. Consistent with our observations, a handful of studies with a small number of cases and a cohort study of 74 patients by Mato et al. concluded that the efficacy of BTKi therapy does not seem to be altered by prior venetoclax treatment [11,37,38,39]. Although sequential resistance to both BCL2 and BTK inhibition associated with dismal outcomes and poor prognosis may occur in some patients, venetoclax plus ibrutinib combination therapy or venetoclax retreatment seem to be promising approaches to address double-class-resistant cases, provided that dual-resistant subclones are absent in the selected patients [21,29,33,40,41,42]. Considering the updated data from the phase 2 CLARITY trial, chemotherapy-free, time-limited venetoclax plus ibrutinib therapy targeting distinct CLL subpopulations can provide synergistic activity in R/R CLL patients, overcoming acquired resistance and conferring complete response rates comparable with other venetoclax-based regimens [43,44].

In summary, our study demonstrates the feasibility and clinical value of the robust detection of the two most common *BCL2* mutations underlying venetoclax resistance in a real-world cohort of patients with R/R CLL. To our knowledge, this is the largest R/R CLL cohort outside of clinical trials to date in which *BCL2* resistance mutations were screened during Bcl2 inhibition. The vast majority of our mutation-bearing patients underwent subsequent disease progression during the follow-up period, and based on the presented data, driver mechanisms of acquired venetoclax resistance may be uncovered in up to 45% of the cases experiencing clinical relapse using a sensitive screening method for only these two aberrations. Although longer follow-up times and prospective studies on independent patient cohorts will be required for a refined assessment of the predictive power of these ddPCR tests, the clinical value of such analyses in the standard-of-care workup algorithms of venetoclax-resistant CLL will likely be increasing in the future. Retrospective interrogation of additional loci in the anti-apoptotic gene *BCL2* revealed multiple, simultaneously detectable mutations with heterogeneous and temporally changing abundance, suggesting a complex subclonal architecture characterized by minor subclones that carry distinct genomic features and cooperatively contribute to venetoclax resistance. Further genome-, epigenome-, and transcriptome-wide studies, preferably coupled with functional readout will be essential to achieve a deepened understanding of key mechanisms orchestrating the interplay between various CLL compartments driving resistance to targeted Bcl2 inhibition.

## 4. Materials and Methods

### 4.1. Patient Samples

Peripheral blood samples from 67 patients (46 males and 21 females) with R/R CLL treated with venetoclax monotherapy or venetoclax in combination with anti-CD20 rituximab were collected from 11 Hungarian oncohematological centers. Fixed-duration venetoclax plus rituximab combination was applied in 23.9% (16/67) of the patients, while time-limited and continuous venetoclax monotherapy was administered in 7.5% (5/67) and 68.6% (46/67) of the cases, respectively. At the time of the last follow-up, 11 patients had stopped the fixed-duration venetoclax treatment after a median of 26 months (7–32 months) due to subsequent deep remission as defined by Hungarian treatment protocols. The cohort represented a pre-treated patient population with a median of two lines (range: one to six lines) of prior therapy. This cohort also comprised a subset of cases with prior ibrutinib therapy investigated within the framework of the Hungarian Ibrutinib Resistance Analysis Initiative, published previously by our research group [45]. Following the ramp-up period, selected patients received venetoclax as a single agent or in combination with rituximab in a daily dose of 400 mg for a median of 20 months (range: 1–83 months).

Samples were collected at the time of disease progression from 23 patients experiencing relapse following a median of 14 months (1–48 months) of venetoclax therapy. In 44 cases with no clinical signs of disease progression, the median follow-up time was 23 months (range: 1–83 months) with samples obtained at the time of the last follow-up. Median 6 follow-up samples (range: 2–11) from 4 patients harboring multiple *BCL2* mutations unveiled by ultra-deep targeted sequencing and droplet digital polymerase chain reaction (ddPCR) at the time of disease progression were collected as part of the routine diagnostic work-up and were analyzed retrospectively. The clinical characteristics of our patient cohort are summarized in Supplementary Table S1.

Peripheral blood mononuclear cells (PBMCs) were separated by density gradient centrifugation following the assessment of leukemic cell purity by flow cytometry using CD5/CD19/CD45 immunophenotypic markers. In cases with low whole blood counts, leukemic cell purity was assessed using CD19/CD5/CD22/CD43/CD79b/CD81/CD3/ROR1 immunophenotypic markers following the most recent European Research Initiative on CLL (ERIC) guideline on minimal residual disease monitoring in CLL [46]. DNA isolation was performed using the High Pure PCR Template Preparation Kit (Roche Diagnostics, Basel, Switzerland) following the manufacturer’s instructions. After DNA extraction, *TP53* mutations and *IGHV* mutation status were assessed according to the most recent ERIC guidelines [47,48]. Chromosomal abnormalities, including deletions of chromosomal regions 11q, 13q, and 17p as well as trisomy of chromosome 12, were analyzed by interphase fluorescence in situ hybridization (FISH) using dual-color Vysis probe sets (Abbott Molecular, Des Plaines, IL, USA). PBMCs from three healthy donors were used as negative controls. Written informed consent was obtained from all participants, the study was approved by the Hungarian Medical Research Council (ID: 45371-2/2016/EKU), and conducted in accordance with the Declaration of Helsinki.

### 4.2. Droplet Digital PCR

Ultra-sensitive screening for *BCL2* G101V and D103Y mutations, as well as additional *BCL2* variants unveiled by next-generation sequencing, was performed by ddPCR using custom assays designed for the quantitative assessment of mutant and wild-type alleles in each altered genomic position (D103E, single tube mutant/wild-type allele assay: dMDS433558996; A113G, mutant allele assay: dHsaMDW3410683143; wild-type allele assay: dHsaMDM3410683141; V156D, single tube mutant/wild-type allele assay: dHsaMDS355891150). Reactions were carried out according to the manufacturer’s instructions on a QX200 Droplet Digital PCR system (Bio-Rad Laboratories, Hercules, CA, USA) with the recommended 90–100 ng of input DNA. Fractional abundance (FA) depicting the allelic burden of the selected variants was calculated from the ratio of the number of detected mutant DNA molecules (a) and the total number of detected mutant plus wild-type (b) DNA molecules (FA = a/(a + b)). FA values were normalized to the leukemic cell purity assessed by flow cytometry (referred to as cancer cell fraction, CCF). The quantitative reliability of the ddPCR assays was assessed with dilution series for each variant and sensitivity was determined based on droplet counts in each sample. The median lower limit of detection proved to be 0.01% as calculated from the final droplet counts.

### 4.3. Targeted Ultra-Deep Next-Generation Sequencing

Targeted next-generation sequencing (NGS) was performed on samples obtained at the time of disease progression using a QIAseq Targeted DNA Custom Panel (Qiagen, Hilden, Germany) utilizing unique molecular identifiers and covering four genes (*BCL2, BTK*, *PLCG2,* and *TP53*) as previously described [45]. Libraries were prepared according to the manufacturer’s recommendations and sequenced on a MiSeq sequencing platform (Illumina, San Diego, CA, USA) using a 150 bp paired-end configuration. Data processing and bioinformatic analyses were carried out with the smCounter2 workflow and variants were annotated using the ClinVar, COSMIC, dbSNP, SnpSift, and SnpEff databases. Additionally, gene-specific databases such as The *TP53* Database (originally the IARC database) and Seshat were used for annotating somatic *TP53* mutations [49,50]. The allelic burden of each mutation defined as variant allele frequency (VAF) was normalized to the leukemic cell fraction measured by flow cytometry.

### 4.4. Statistical Analysis

GraphPad Prism 9.1.0 software (GraphPad Software, San Diego, CA, USA) was used for calculating the median values and for performing the Mantel–Cox test to compare progression-free survival (PFS) and overall survival between patients harboring *BCL2* resistance and cases with wild-type *BCL2* G101 and D103.

## Data Availability

The data presented in this study are available on request from the corresponding author.

## Supplementary Materials

The following supporting information can be downloaded at: https://www.mdpi.com/article/10.3390/ijms24065802/s1.
