# Supplementary material for: Landscape of BCL2 Resistance Mutations in a Real-World Cohort of Patients with Relapsed/Refractory Chronic Lymphocytic Leukemia Treated with Venetoclax
Source: Int J Mol Sci. 2023 Mar 18;24(6):5802. doi: 10.3390/ijms24065802 (PMC10058128; doi:10.3390/ijms24065802)
Supplement: Supplementary file 1 [file ijms-24-05802-s001.zip › Kotmayer et al Suppl. Figure S1_2023.pdf]

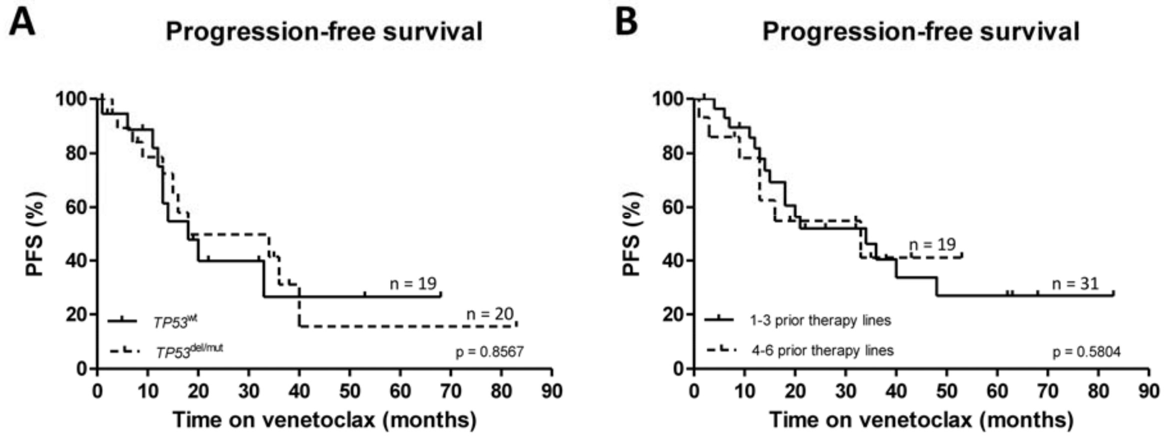

Figure S1. Progression-free survival (PFS) and overall survival (OS) of relapsed/refractory CLL patients receiving continuous venetoclax therapy. (**A**) No significant difference was observed in patients with TP53 disruptions (deletion of 17p and TP53 mutations) compared to cases with wild-type TP53. (**B**) The number of prior therapy lines (1-3 vs 4-6 lines) did not influence the PFS in CLL patients with continuous venetoclax monotherapy.
